# Efficacy of Calomel in Large Doses in Typhus Fever Aided by Cold Affusions

**Published:** 1845-02

**Authors:** Joshua Burgess

**Affiliations:** Surgeon, London


*Efficacy of Calomel in large doses in Typhus Fever aided by Cold Affusions. By Joshua Burgess, Esq., Surgeon, London.* Typhus is the typification of disease of membranous tissues, beginning with the pituitous membranes: the serous and mucous linings of the large cavities; the mucous linings of the alimentary, gastric, and intestinal canals; the epithelium; and, above all, the skin, the extensive covering of the surface of the body; all being more or less implicated, directly or indirectly, in the progress of typhus.

The great extent of membranous tissues, beyond all others, in the animal organization, their important and almost universal function, and their singular sympathy in health, disorder, and disease, sufficiently explain the inveteracy of the character of typhus.

I am induced to make these remarks prefatory to attempting to explain the *ratio medendi* of calomel and cold affusion in the treatment of typhus, a few observations upon which I submitted to you in your number of the 14th of September.

Every form and degree of membranous inflammation is attended with the phenomena, and characterized by some of the symptoms, of typhus, and it is an interesting inquiry, if disorder, or irritation only of membranous tissues, be not accompanied by mitigated symptoms, which range under the phenomena of typhus, excepting perhaps those cases in which the spinal axis and ganglionic periphery of the nervous system are implicated, and when the vascular system becomes secondarily and partially disturbed.

All the exanthemata have the typhus type.

If it be assumed, as above, that the great mucous surfaces, including the skin, so universally expanded over the external and internal parts of the animal structure, in and through which transpiration, exhalation, absorption, secretion, and sensation, are so wonderfully performed, are the seats of disease in typhus, the remedial agency of calomel and cold affusion is not difficult of solution.

The skin, including the epithelium, being, as above stated, the most extensive tissue in the human organization, important results must attend the derangement of its functions, whether exhalent or secreting, death speedily resulting from that condition, which Dr. Willis has graphically described as producing over the surface "an impervious glaze."*

The slimy secretions with which the gastric and intestinal canals are congested, impair or destroy the functions of digestion or assimilation; the condition of the skin, covered with a clammy and tenacious gum; the sordes plugging up the cavities of the fauces; internal and external ears, eyes, nose, lips, and even extending over the nails and hair roots—all combine to produce the characteristic and formidable symptoms of typhus.

I will not stay to discuss the proximate or remote causes of typhus, which would be irrelevant to the present inquiry; nor say in what locality or region the disease originates, as the question at present turns upon a *status quo*, upon circumstances and conditions assumed, and which will afford sufficient for present discussion, although I am prepared at the proper opportunity to discuss these points.

If I may be permitted to use a rather fashionable expression, I should say, the reflex action of typhus is cerebral. I consider this to be the fact, since the cerebral symptoms are never entirely relieved till either the skin or the bowels are sufficiently and amply acted upon, use what topical means you may.

The access and progress of typhus is slow, and the predisposition in the constitution must be strong, and of long duration; and the last has probably more influence than any contagious influence whatever. The elements of disease have been accumulating through the media of bad or low diet, bad clothing, intemperance, exposure, malaria, anxiety,

> "——— and the thousand natural shocks
> That flesh is heir to,"

till some slight and accidental disturbance, either gastric, hepatic, intestinal, or cerebral, calls them into action, and the whole train of phenomena which constitutes the typhus type becomes developed. In the milder cases, the febrile paroxysm

---

* Vide "Medical Gazette," July 12, 1844: On the Special Function of the Skin, by Dr. Willis.

is of the remittent character, in its worst forms; when it becomes continued, the arterial, venous, capillary, and exhalent vessels are highly congested; and only that the indistinctly marked stages of rigor or collapse are succeeded by febrile accessions of an intense character, the state of comatose lethargy so characteristic, may be justly compared to a living death.

In this condition, when the vital powers have become nearly subdued, neither mild nor temporizing remedies will rouse the slumbering functions, an impulse must be speedily obtained, directly, through the vascular system, and indirectly, over the ganglionic and cerebral functions; this is best obtained by large doses of calomel, aided and succeeded by cold affusion.

Calomel, in large doses, acts proximately as a sedative, without losing any of its well-known valuable qualities as an alterative, upon the absorbent and glandular structures. The first good it produces is sound, calm, undisturbed sleep, to which the patient has been long a stranger, during which the ganglionic, absorbent, and glandular systems are silently invaded by its alterative agency; the intestines become filled with secretions, ready to be removed by any mild cathartic, (*e. g.*, small doses of neutral salts,) exhibited periodically and regularly, to make them patent; the dose of calomel should be renewed every night, (without any other medicine, only the neutral salts, in solution with infusion of senna and the mindererus' spirit,) and repeated till the functions approach their normal state. The cold affusions over the naked surface of the body are to be practised daily, by throwing over it, while in a sitting posture, a bucketful of cold water, during the acme of the febrile paroxysm.

The breathing becomes more free, and the stertor, so frequently present, gradually subsides; the pulse, from having been oppressed and feeble, yet quick and wiry, becomes compressible, free, and bounding, increased in volume, and diminished in frequency; the peculiar expression of countenance, now collapsed, anxious, and cadaverous, with a cold, lead-like, lack-lustre expression, the eye injected, and the sardonic grin and incoherent mutter quivering on the lip, also subside; the subsultus tendinum and picking of the sheets also cease, and a profuse sweat pervades the surface, the skin becoming relaxed, soft, and elastic, and its temperature gradually restored to a uniform healthful and natural condition.

If cold affusion be supposed to have no specific agency, which I nevertheless think it possesses, in stimulating and strengthening the capillary and exhalent vessels, and the vascular and nervous systems, thus giving tone to the function of the heart, equalizing the circulation, and removing congestions, it at any rate possesses that quality which has been already so successfully excited in the gastro-enteritic systems, of washing away and effectually removing morbid secretions, of opening the mouths of the exhalents, and restoring transpiration, so long interrupted, and which very probably has acted as a remote cause of disease.

One of the phenomena attending the results of this practice is the wonderfully increased action of the absorbent system; the absorbents appear to become rapacious and greedy; and when, from their previously impaired state, effusions or morbid deposits in the tissues have taken place, this increased action becomes singularly developed in their rapid removal.

The ascaris lumbricoides very commonly infests the intestinal and alimentary canals in typhus, as also in the infantile fever of children, when the slimy secretions are in both instances characteristic of the attendant disease, and in both of which their expulsion, or creeping away, is the harbinger of recovery.

The mercury remains too short a time in the system to produce any specific irritation, or hypercatharsis; the intestinal canal, having long been torpid and insensible, renders the calomel comparatively inert as a cathartic, covered as it and the whole of the alimentary passages are with slimy mucus, and its sedative influence on the ganglionic system passively producing profuse secretions, the passages become a vortex, rendered potent by the Epsom, Glauber, or other neutral salts, the natural sensibility of the passages being only perfectly obtained after the necessity has ceased for the administration of the large doses of calomel, and the whole quantity of the calomel taken has been detected again in the stools, ("partly as sulphuret of mercury,") and from its difficulty of solution no doubt can exist of its being in this state wholly rejected in the stools, although in its progress it has produced decided and important results.—*Bulletin of Med. Sci.*, from *Lon. Lancet.*

